# The Treatment of Gonorrhea and Gonorrheal Pelvic Disease in Women

**Published:** 1893-03

**Authors:** C. C. Frederick

**Affiliations:** Obstetrician and Gynecologist to the Buffalo Woman’s Hospital


### THE TREATMENT OF GONORRHEA AND GONORRHEAL PELVIC DISEASE IN WOMEN.

By C. C. FREDERICK, B. S., M. D.,

Obstetrician and Gynecologist to the Buffalo Woman's Hospital.

The topic assigned to me for discussion was The Treatment of Gonorrheal Pelvic Disease in Women. To make the subject more complete, I have considered, in addition thereto, The Treatment of Gonorrhea in Women.

Vaginitis is not so common in the gonorrhea of women as is generally supposed. The more frequent expression of gonorrheal infection is in the cervix and endometrium, especially the cervical canal. The course is not so universally acute as in the male. Often a woman may contract a gonorrhea which pursues a slowly insidious course from the cervix till the whole mucous tract of the genital canal is involved, and never have had a vaginitis.

The treatment of acute vaginitis must be as active as the tolerance of the inflamed canal will permit. Cleanliness and rest are of first importance. The patient should go to bed and remain there during the acute stage. Cleansing of the inflamed tract is best secured by frequent hot water douches. Often only a slowly-running stream of tepid water can be borne. But care in manipulation and persistency in the frequent cleansing will soon establish a tolerance to more active measures. As soon as the patient will bear it, I like to follow the hot douche with a tepid fifty per cent. solution of peroxide of hydrogen, or a ten per cent. solution of sulphate of zinc, or both, using the zinc last.

I know that many of the text-books advise against vaginal injections, giving as a reason therefor that the cervix and endometrium are more liable to infection in consequence. This I do not think can be substantiated by experience. If the uterine canal is not infected by continuity of mucous surface, then injections, which serve to wash away from the external os the swarms of gonococci, will certainly not serve to do it.

As soon as the vagina will tolerate it, a cylindrical speculum should be introduced, after a vigorous douche of water and peroxide of hydrogen, and all mucus removed with cotton swabs. Then pour into the speculum, at least, half an ounce of a ten per cent. solution of nitrate of silver. Gradually withdraw the speculum, thus allowing the silver solution to come in contact with the whole mucous surface of the vagina and vulva.

Neisser, who discovered the gonococcus, affirms that nothing kills this organism as well as a solution of silver nitrate strong enough to coagulate the contents of epithelial cells.

A cervical or endometrial gonorrhea, which has not yet extended to the tubes or pelvic peritoneum, if it be diagnosticated as such, should not be given time in which to complete its awful work. It may lie partially dormant there till something arouses it to activity again, when it may light up a pelvic peritonitis, salpingitis, and ovaritis.

To treat such a condition properly, the patient's cervix must be dilated and the whole uterine tract curetted. Some use rapid dilatation under an anesthetic. I prefer to use slow dilatation, with aseptic laminaria tents. To do this operation, and have it successful, there are three principal points to attain. It must be done aseptically; it must be thoroughly done; free drainage of the uterine cavity must be secured for several days. Dilatation by aseptic laminaria tents, under full aseptic precautions, is as safe as any operation in surgery. The dilatation is slow, but it is thorough, giving an abundance of room in which to work, and, furthermore, the rugosities of the cervix are smoothed out, so that the curette can more effectively remove the cervical epithelium. The free drainage, after the operation, is secured by the thoroughness of the dilatation and the slowness with which the cervical tissues contract. I do not believe that this can be well done by rapid dilatation. I introduce a tent, as large as the cervix will admit, and allow it to remain from eighteen to twenty-four hours, then remove this and put in another as large as can be introduced. After removing the second tent under anesthesia, if the canal is not fully dilated, I usually complete the dilatation with the Hegar's hard-rubber graduated uterine bougies. Then thoroughly curette the uterine and cervical canal, wash out the *débris*, dry the cavity with sterilized absorbent gauze, and inject it with a ten per cent. nitrate of silver solution. Remove the excess of the silver solution and pack the cavity with strands of iodoform gauze, which may remain for from twenty-four to forty-eight hours. The patient should remain in bed for one week or more, the vagina kept clean, and the bowels free. If for any reason it be thought advisable, the uterine cavity may again be packed with iodoform gauze after removing the first dressing.

Gonorrheal inflammation of the pelvic organs of woman is either acute or chronic, from the therapeutic standpoint. The treatment of the acute stage varies little from that of pelvic inflammation due to other causes. This is essentially expectant in the vast majority of cases. Rest in bed, hot applications, and anodynes as required, constitute the routine treatment. Caution should be exercised in the too free administration of opium, since this inhibits intestinal peristalsis, thus favoring adhesions of the bowel. In nearly every abdominal section for tubal or ovarian disease, having a history of previous inflammation, there are adhesions of omentum or knuckles of gut to the diseased appendages. If less opium were given, and the bowels made to act daily, fewer bowel adhesions would probably result. Free catharsis frequently relieves pain in these cases, and a loaded bowel sometimes makes the pain worse. If opium be given liberally at the outset, the patient will invariably cry for more anodyne the instant she feels the slightest pain returning. In all probability, most of these patients would be comfortable with less opium than is generally given.

If pus forms and it can be evacuated and the sac drained through the vaginal vault, such a course is expedient. When the abscess is healed, the patients are not always cured; in fact, they are seldom cured. If pus is present and cannot be evacuated through the vaginal vault, abdominal section is necessary, as acute abscesses are more prone to point and rupture than are those of the chronic stage. A general peritonitis succeeding to a pelvic peritonitis, at however short an interval, demands immediate section, for a leaking pus sac, somewhere in the pelvis, usually is the cause of the general peritonitis, and must be taken out at once if the patient's life is to be saved.

Because a woman has acute pelvic disease of gonorrheal origin, is no positive reason why she should not entirely recover. Such cases I have seen, but they are but a small percentage of the entire number afflicted. The usual sequel is general pelvic adhesions with pus in tubes or ovaries, or in both. Many patients who rise from their sick bed apparently well, and whom we pronounce recovered, are not recovered. One, two, three, five, or ten years after the acute attack, their history of suffering during these years tells that they were not recovered. We all remember patients whom we thought well, who in the light of later developments proved the reverse. How many more, of whom we lose sight in the flight of time, repeat the same story.

The chronic stage of the disease is worse than the acute. Pain, menorrhagia, dysmenorrhea, sterility, chronic invalidism, is the picture. If you record her temperature for a week, the curve will probably be indicative of pus. Manifestly a local condition which will drag a woman to such depths of invalidism and suffering is not amenable to palliative treatment, else the *vis medicatrix naturæ* would have restored her to health.

If there be pus in her pelvis, nothing will help her till it is removed. Some non-operating gynecologists, in a spirit of conserva tism, criticize those who do operate for their boldness in removing pus tubes and ovaries. They would consider it less heroic and more conservative to puncture and drain these when possible through the vagina. It seldom happens that a pus tube or a tubo-ovarian abscess can be evacuated through the vagina. When they are so treated, they are generally slow to heal, and sometimes a fistula remains for years. Another argument against this procedure is that tubal and ovarian abscesses are generally multiple. Unless all of them are drained, it would be useless to open one. I here present for your inspection some specimens of pus tubes and ovaries which I have recently removed, all of which contain multiple abscesses. One, especially, seemed to be most favorable for puncture and drainage, as the largest abscess lay in Douglas' pouch, and was adherent there. In this same patient, however, there were three other pus cavities which such a puncture would not have drained, and section would have been a necessity to cure her, and undoubtedly the fistulous track of the puncture would have complicated the operation.

To remove the pus will save the patient's life ; but will simple removal of pus, restore her to health if the tubes and ovaries are left covered and pressed upon by ever-contracting adhesions ? By removing the pus the element which threatens her life is eliminated. The woman may be much improved by being rid of the constant poisonous influence of its presence. But she will continue to have pain, irregularities of menstruation, and a certain degree of chronic invalidism so long as the uterine appendages are involved in dense adhesions. Leaving out of consideration the pain and menstrual difficulties, there must be transmitted through the great sympathetic nervous system a profoundly depressing effect upon the innervation and vital functions of the individual. If this position is tenable, then we cannot be content with the evacuation of pus when present, even if it were always possible. We must remove the diseased and constricted tubes and ovaries. Sometimes it is necessary to fairly dig them out of their dense lymph investment. Occasionally it is impossible to remove them, but rarely. In such an event, if pus cavities exist, they should be raised and sewed to the abdominal opening ; or, if that is impossible, they should be evacuated, cleansed, packed, and drained. In all sections where pus cavities have ruptured or leaked during operation, the abdomen should be thoroughly flushed and a drainage-tube inserted.

The endometritis which existed prior to, or was associated with, a salpingitis, generally needs attention after the patient has recovered from the abdominal section. I think a neglect of this has not infrequently retarded or prevented the patient's complete restoration to health. It is always safe practice in such cases to curette the uterus, thus insuring a healthy endometrium and a complete involution of the uterus.

Right here arises a pertinent question: Do all women entirely recover their health whose diseased appendages are removed, and who survive the operation?

Their lives have been saved; the causes which wrecked their health are no longer operative. Do they again return to perfect health and usefulness? In most instances they do, while others do not. Is it not asking too much and expecting too much from a poor wrecked body and nervous system which have borne the strain of years of suffering, to at once regain their former vigor, because the source of all the mischief is, at so late a date, removed? As well might we expect, after the fire has been quenched, that the building which it has just destroyed should again rear itself in all its former beauty and symmetry. The longer a woman carries the causes of her invalidism, the more profound and irremediable will it become, always taking into consideration the vitality of the individual and her powers of recuperation.

Operation should be supplemented by Weir-Mitchell's rest treatment in most cases of profound invalidism. This does much to restore nervous equilibrium, build up the blood, improve the general nutrition, and store up nervous and physical energy. When this has been accomplished, the benefits of the operation will become manifest.

The earlier operation is resorted to in pelvic disease, the better will be the woman's chances of complete recovery. If her general health has not been undermined, she will promptly get well. Operation should not be the last resort. If the family physician recognizes the conditions present, his duty to his patient is not to let her wait, but to urge her to do that which is for her best interests. Until within the last decade all abdominal operations were done as a last resort. Many lives have been saved, and much suffering prevented, by the surgeons advising early operations. Still, there are those who wait too long. Procrastination here is not only a thief of time, but also the destroyer of one of the great blessings which goes to make life worth living—health.

DISCUSSION.

Dr. DeLancey Rochester: I am very glad that this subject has come up for discussion, for I think it is one of vital importance to the community. If the statements of the gynecologists are correct, and I believe they are, the question of the marriageability of a man who has once had gonorrhea is one of the greatest import. I believe that if a man has once had gonorrhea his urethra is rarely, if ever, in a healthy state afterward, and always carries with it the possibility of infecting any woman with whom he may have intercourse. Under these circumstances, I think it is worse than foolish,—I think it is *criminal*, for a man to marry. Therefore, I think it is our duty as physicians to frankly and fearlessly tell those who may consult us, that if they have ever had gonorrhea, they ought never to marry. I think, Mr. Chairman, that if the young men were *early* informed of the dangers of this serious disease, which is so frequently dismissed as "only the clap," there would be much less of it in the community, and the objects of the "social purity league" would be more rapidly attained by an appeal to the physical man to keep himself healthy for the sake of the physical well-being of the woman whom he may make his wife.

Dr. H. E. Hayd: The increasing frequency of this terrible disease makes our responsibility to our patients, and to society in general, a very serious matter. It is a disease which is not confined to the poor, but is peculiarly common in the better walks of life. In so far as we intensify its importance, and keep before the public its terrible possibilities, we are doing a grand work. But when we go so far as to say that clap in the male is an incurable disease, we assume a position unscientific and equally dangerous. That a young man should be blighted and forswear matrimony because he was the early victim of the clap, is absurd. No doubt, a very few cases remain uncured, but compared to the total number once diseased, the proportion must be very small indeed; because the percentage of men who have at one time in their lives had the clap is so great, that at least half of the women in the land would be the victims of salpingitis, pyosalpingitis, and ovarian abscess. Therefore, I maintain that after proper treatment and some months of entire absence of discharge, and taking the precaution to sound the urethra to relieve it of any hypersensitive patches and strictured areas, a man may marry and should marry, as discharging a duty he owes society.

I believe it was a very dangerous and sweeping conclusion that Noeggerath assumed in his cases, and I am satisfied that he did not—because he could not—eliminate all the other causes of pelvic mischief. Did he include the colds during menstruation, the repeated catarrhs, the result of our careless and forced methods of living, the dietetic social and sexual excesses, the needless and too often dangerous minor gynecological operations, and last, but not least, the terrible practices that so many of our best women in society resort to, to thwart maternity. I admit the terrible frequency of gonorrheal endocervicitis, endometritis, and salpingitis, in our poor, unfortunate, and trusting wives, but I will not admit that the gonorrhea antedating marriage five, ten, and fifteen years is responsible for the pathological conditions we see, so long as so many other frequent sources of their production exist.

Dr. Mann called attention to a very important fact, and that is, how easily gonorrhea is overlooked in the female. I have repeatedly seen cases of undoubted cervical clap, and yet saw no vaginal discharge. Again, it is equally true that these latent and creeping gonorrheas of Noeggerath and Sinclair are the cause of the greatest amount of mischief, because a woman is often hopelessly invalided before she is conscious of the existence of any trouble. Be on the lookout for them, and put them to bed early, and you will be surprised to see how many and how often they quickly get well.

Another point in Dr. Frederick's paper which impresses itself upon me the more I see pelvic trouble: I am of the opinion that vaginal puncture should be done only when the patient is moribund, and, therefore, could not stand a radical operation, because we deceive ourselves by opening up one abscess and expect a cure when there remains three or four others simply separated from one another by a thin partition.

The President: Though the hour is late, I trust the society will tolerate a word or two from the Chair, in relation to this important subject.

The three papers that we have listened to cannot but prove instructive, and are a valuable contribution to the subject of gonorrhea in women. Some three or four years ago, in reviewing William Jap Sinclair's monograph, entitled Gonorrheal Infection in Women, I ventured to remark that the literature of this disease must be entirely rewritten, and the teachings of the schools entirely recast, in view of the awful consequences of neglect to entirely stamp out or prevent the propagation of this subtle, deceptive, and virulent malady. I further called attention to the fact that it was no perfunctory task to treat a case of gonorrhea in its acute stages. It has been treated heretofore altogether too indifferently, and in a light or trifling manner; physicians contenting themselves for the most part with ministering to the first distress, paying little heed to the secondary possibilities of the infection. At that time I also ventured to state that a woman who accepts offer of marriage finds herself treading upon dangerous ground, and that it becomes a problem of serious import when her answer may fetch her to a spring of woes unnumbered.

On another occasion I ventured to state that no contract of marriage should be made unless a competent physician had determined that there was no danger of infection on the part of the male. These seemed to be somewhat exaggerated or advanced views at the time they were offered, but I am glad to note that the trend of the papers we have just listened to is in the direction of the arguments that I have heretofore advanced.

It is to Noeggerath and Neisser that we are indebted for the original research that has led to a newer pathology on the subject in question. Before Noeggerath published his Opuscule in 1872, we were much in the dark, and it has taken something like twenty years of continual hammering to rouse the profession to the dangers that lurk in an uncured or latent gonorrhea that Noeggerath then pointed out.

When Neisser announced his discovery of the gonococcus, it seemed to furnish a confirmation of Noeggerath's theory, and these two must ever go hand in hand as the culminating point of the new pathology which has not been successfully challenged by any competent authority.

The society, I am sure, feels very much indebted to the essayists who have so succinctly, and yet so pointedly, presented this subject, and I trust that the group of papers and the discussion will be offered to the BUFFALO MEDICAL AND SURGICAL JOURNAL for publication, so that they may be preserved for reference and study.

Dr. J. H. PRYOR: A part of this discussion has seemed to me unscientific. We are considering a medical topic, not a moral one. The term gleet has been carelessly employed, and it becomes necessary to decide when a urethral discharge is infectious or not. The fact that a man has a discharge may mean much or little, dependent upon the character of that discharge, and the conclusion that it is dangerous and wrong for him to marry is hasty and unwarrantable. To my mind, this matter has been much exaggerated, and some of the radical opinions stated here today cannot be supported by clinical observation. We can only say, at present, that the question hinges on the presence or absence of the gonococci. I have risen to make a few remarks in reference to an experience gained in Germany, where investigations were being prosecuted to verify the work of Neisser. The points for detection are repeated examinations of the discharge, and sometimes producing an exacerbation by irritating injections. The importance of recognizing cocci in the pus cells, and remembering that they are not round, but somewhat crescentic (called by the Germans semmel-shaped), and always in pairs. It is sometimes difficult to decide between them and other forms, and they often exist in such small numbers during the chronic stage of gonorrhea as to elude detection. When the discharge is made up largely of mucus and epithelial cells, with few pus cells, there is usually present only a mild catarrhal condition tending to recovery. If the morning drop is a clap shred, there is usually a localized granular patch. Gonococci may or may not be present. We must be sure to bear in mind that chronic gonorrhea, especially of the deep urethra, may be present for an indefinite time and not be manifested by any discharge whatever. In these cases, and they are quite common, it would be very superficial to place any dependence upon the absence of discharge.

In every suspected case, then, we should examine the morning urine for clap shreds. While working in Gerhard's laboratory, in Berlin, his assistants and I found gonococci in these shreds, several times five years after an apparent cure, and in two cases, eight and ten years after the cessation of the acute attack. So far as I know, the results obtained by VanVoorden at this time, have never been published. They were very interesting, as showing the chronicity of gonorrhea, and the long life of the gonococci in the deep urethra after all symptoms had disappeared. Gerhard directed the examinations with the idea of proving a conviction that the gonococci were quite generally causative of rheumatism, and this led to the results just mentioned. It is not an easy matter to decide when it is safe for a young man who has had gonorrhea to marry. I disagree most emphatically, however, with some of the views presented today. There are scientific methods for settling the question, and no need of sweeping generalizations. I believe that any discharge is suspicious until it is known to be innocent, and we can only determine this fact by repeated and careful examinations extending over a long period. Every clinician could, after reflection, describe cases which support both sides of this controversy, but we can gain little by basing opinions on inexact data. When we speak of gleet, let us know what the term means, and I, for one, ask the right to doubt any statements that a case of pyosalpinx is due to gonorrhea in a husband, unless the pus is known to contain the specific germ. That such infection occurs frequently, there seems to be no ground for doubting, but the tendency to ascribe certain conditions to one cause has been noticed before in the history of medicine, and, perhaps, in time more conservative ideas will prevail in regard to this very important and interesting subject.

---

Hydrochloric Acid in Vomiting.—Dr. Alkiewicz mentions that in various kinds of vomiting he has found great benefit from small and frequent doses of hydrochloric acid—well diluted, of course. In one case of the vomiting of pregnancy, where none of the ordinary remedies had any effect, hydrochloric acid proved successful, though it had to be given for a fortnight before it entirely arrested the sickness. In more than ten cases of cholera nostras in adults, with vomiting, hydrochloric acid was given with good results. Again, where vomiting was due to acute dyspepsia from errors in diet, and where it occurred in the course of influenza, scarlet fever, or other contagious diseases, the same remedy proved equally efficacious.— *Occid. Med. Times.*

